# Research on the application of computer-assisted surgical technology in ophthalmic plastic surgery education​

**DOI:** 10.1186/s12909-025-08158-8

**Published:** 2025-12-22

**Authors:** Junming Li, Jing Li, Haiyan Zhao

**Affiliations:** 1 School of Computer Science and Engineering, Computer Science and Technology, Tianjin University of Technology, Class 1, No. 391, Binshui West Road, Xiqing District, Tianjin, 300384 China; 2 Teaching and Research Section of Medical Imaging, Medical Technology Department, Haiyuan College, Kunming Medical University, No. 389, Haiyuan North Road, High-tech Development Zone, Kunming, Yunnan 650106 China; 3 The First People’s Hospital of Yunnan Province, The Affiliated Hospital of Kunming University of Science and Technology, Department of Ophthalmology, No. 157 Jinbi Road, Kunming, Yunnan 650032 China

**Keywords:** Computer-assisted surgical technology, Three-dimensional image analysis, Teaching reform, Ophthalmic plastic surgery, Orthanc

## Abstract

**Background:**

This study explores the use of Orthanc, Mango, and 3D printing technologies to simulate ophthalmic plastic surgery teaching for undergraduate medical students. By incorporating 3D image modeling, segmentation, surgical manipulation, quantitative measurement, and virtual surgical planning, we aim to provide an intuitive, visual representation of complex surgical procedures and enhance students’ theoretical knowledge, diagnostic skills, and surgical competence.

**Methods:**

Fifty-eight clinical medicine students from the 2022 cohort at Haiyuan College, Kunming Medical University, were randomly assigned to an experimental group (computer-aided teaching) or a control group (conventional teaching). Teaching effectiveness was evaluated through theoretical exams, practical assessments, and satisfaction surveys over one semester.

**Results:**

The experimental group showed significantly higher scores in theoretical knowledge, practical surgical skills, and teaching satisfaction (all *P* < 0.001), with large effect sizes (Cohen’s d > 0.98). The satisfaction rate in the experimental group was 91.24%, nearly 10% points higher than that in the control group (81.31%).

**Conclusion:**

In this study, the computer-assisted surgical technology was associated with a simplified teaching process, improved spatial understanding among participants, and led to a significant enhancement in students’ mastery of ophthalmic plastic surgery as measured by our assessments.

**Supplementary Information:**

The online version contains supplementary material available at 10.1186/s12909-025-08158-8.

## Introduction​

Ophthalmic plastic surgery education faces two major challenges: the complex anatomy of the orbit is difficult to convey through two-dimensional images or static models, and clinical training opportunities are limited by ethical and resource constraints. Only 17.3% of medical schools offer undergraduates the chance to observe complex procedures such as orbital fracture repair [1]. Traditional simulation methods—such as cadaver dissection or silicone models—are costly, difficult to scale, and often unavailable, which in turn leads to approximately 43% of graduates lacking confidence in designing orbital repair plans [2]. CAST (Computer-assisted surgical technology) has become integral to modern surgical practice, supporting preoperative planning, intraoperative navigation, and postoperative assessment. Its application in medical education is increasingly recognized as a key area of instructional innovation [3, 4]. Studies highlight the value of AI-enabled virtual simulation in improving surgical skills [5, 6], and 3D visualization based on CT (Computed Tomography)/MRI (Magnetic Resonance Imaging) data has been shown to enhance the precision and effectiveness of surgical training [7–10]. This study explores the application of CAST in ophthalmic plastic surgery teaching, aiming to overcome the drawbacks of traditional teaching models through improved teaching methods, enhance teaching participation, stimulate students’ curiosity, and cultivate innovative thinking. To address this gap, we developed an end-to-end workflow using open-source tools, from DICOM (Digital Imaging and Communications in Medicine) image acquisition to surgical simulation, aiming to improve teaching quality in this specialized field.

Ophthalmic plastic surgery focuses on restoring periocular function and aesthetics, encompassing procedures like blepharoptosis correction, orbital fracture repair, and eye socket reconstruction. CAST has improved surgical safety and predictability through high-resolution image reconstruction, real-time navigation, and precise manipulation, with established guidelines for its use in these contexts [11–13]. Numerous studies have demonstrated its value in both clinical practice and educational settings. However, existing literature lacks detailed methods for integrating CAST into undergraduate courses. This is particularly challenging for undergraduate education, which often involves large student cohorts. Furthermore, conducting clinical surgical training in actual operating rooms is frequently impractical due to venue limitations and ethical constraints. To address this gap, we employ Orthanc, Mango, and 3D printing technology [14, 15]. The first two are free, open-source software platforms with user-friendly installation and standalone operation capabilities, ideal for educational purposes. Together, they enable seamless retrieval of patient CT/MRI DICOM data from PACS (Picture Archiving and Communication Systems) systems, followed by 3D modeling, image analysis, and 3D printing file generation on personal computers [16], thus providing end-to-end simulation of ophthalmic plastic surgery workflows at a reduced cost. Applications of CAST in medical education have evolved from early 2D image analysis to full-process 3D simulation [17]. In recent years, deep learning-based image segmentation techniques (e.g., The U-Net model) have improved the segmentation accuracy of orbital bones and soft tissues to 95.6% [18], providing a morphological basis for surgical simulation. VR (Virtual reality) technology has been shown to increase surgical step accuracy by 28.7% in ophthalmic training [19], but high equipment costs (US$50,000–80,000 per VR system) limit its educational accessibility. In contrast, open-source software, e.g., Orthanc and Mango combined with 3D printing offers a low-cost alternative for resource-constrained institutions [20]. Studies show that 3D-printed orbital models can improve anatomical structure recognition speed by 40%, with operational costs 1/20th of traditional VR systems [21].

Although CAST has formed standardized protocols in clinical surgical planning (e.g., AAO and ESOPRS guidelines), its systematic application in undergraduate education faces three major gaps: ① Lack of teaching process design tailored to undergraduate cognitive characteristics, with existing research focusing primarily on resident training [22, 23]; ② Insufficient research on the educational adaptability of open-source tool-chains, especially in DICOM data processing and teaching model conversion; ③ Lack of large-sample controlled studies validating teaching efficacy. This study constructed a closed-loop teaching system of “DICOM data acquisition–3D modeling–virtual surgery–3D printing”. Through a randomized controlled trial, we explored its potential to enhance the theoretical and practical competencies of undergraduate medical students, hoping to contribute to the ongoing discourse on addressing these research challenges. By implementing this system in teaching, we aim to enhance students’ understanding, memory, and mastery of surgical procedures. The selected tools enable direct 3D modeling and simulation of DICOM-formatted medical images, effectively replicating hospital PACS systems in clinical settings. Their portability and standalone functionality allow independent deployment in clinical departments or classrooms, meeting the needs of group teaching and alleviating the inherent conflicts between clinical education and healthcare operations in traditional large-scale training.

## Methods​

**Fig. 1:**
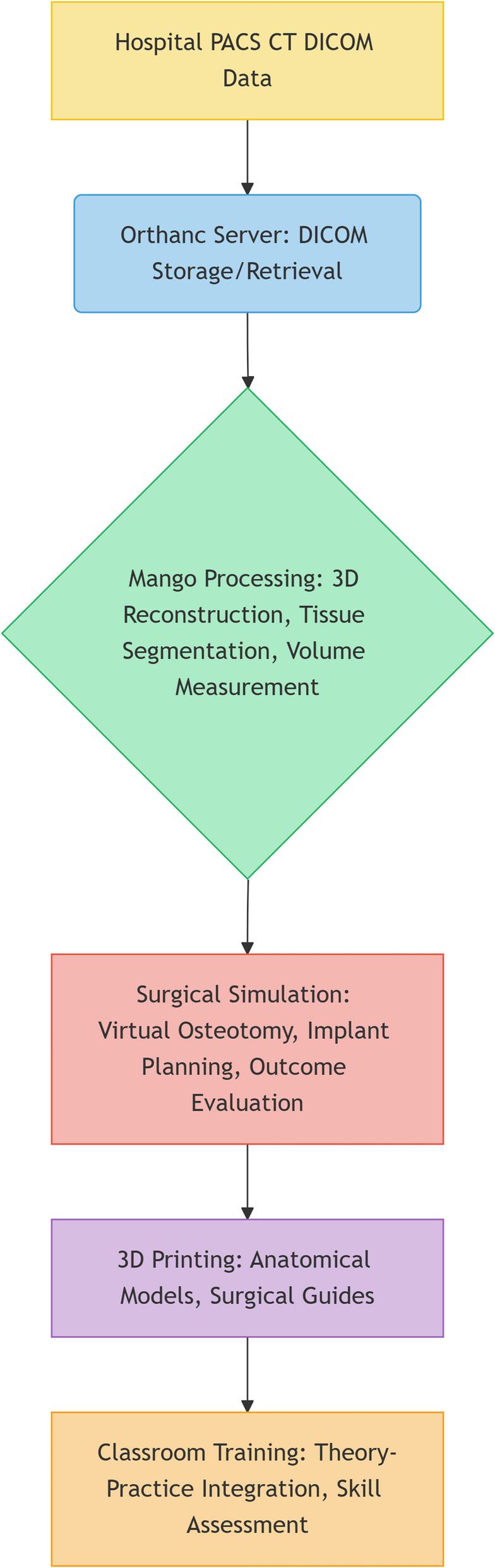
Computer-aided surgical training integration workflow diagram combining Orthanc, Mango, and 3D printing technologies

We developed an end-to-end simulation pipeline (Fig. 1) consisting of four stages: DICOM retrieval via Orthanc, 3D modeling using Mango, virtual surgical planning, and 3D model fabrication.

### Experimental data​

CT datasets from two patients were obtained from the Ophthalmology Department of The First People’s Hospital of Yunnan Province, detailed as follows:​Case 1: Male, 48 years old (CT number: 2183162), scanned on 2025-01-10. Dataset characteristics: 142 slices, 1 mm slice thickness, 512 × 512 pixel resolution, 216 × 263 mm field-of-view (FOV).​Case 2: Female, 59 years old (CT number: 2185331), scanned on 2025-01-12. Dataset characteristics: 145 slices, 1 mm slice thickness, 512 × 512 pixel resolution, 200 × 200 mm FOV. The study was approved by the institutional review board (ID: 2025-GN009).

### Software tools

Orthanc and Mango are free, open-source software platforms with publicly available source codes:

Orthanc v1.12.0.9 (Release: 2023-12-15, https://www.orthanc-server.com/download.php) was deployed for DICOM data handling, enabling storage, retrieval, transmission, and printing of medical images. It mimics hospital PACS systems and supports standalone operation.

Mango v1.0.0 (Release: 2024-03-22, https://www.huajunxiazai.com/soft/288032.html), The 3D visualization tool with key functionalities including:3D Reconstruction: Imports CT/MRI DICOM files and allows operations like tissue segmentation, automatic bone removal, and bed plate elimination [22]. Editing & Analysis: Provides tools for 3D anatomical measurement, linear perspective visualization, and region-of-interest segmentation [24]. Clinical Integration: STL (Stereo lithography) file generation for 3D printing, multi-angle lesion observation, and surgical planning (Windows installer: 11.6 MB). These tools collectively enabled end-to-end processing for major ophthalmic procedures (orbital fracture repair, blepharoplasty, eye socket reconstruction, periocular tumor resection) [25].

### Experiment design

A Windows-based personal computer (specifications: Intel Core i5-1155G7 CPU @ 2.50 GHz, 16GB RAM, Windows 11 Home, HUAWEI) was used to install Orthanc and Mango for surgical simulation teaching, demonstrated via orbital fracture repair and eye socket reconstruction cases.​.

### Data acquisition and preparation​

**Fig. 2:**
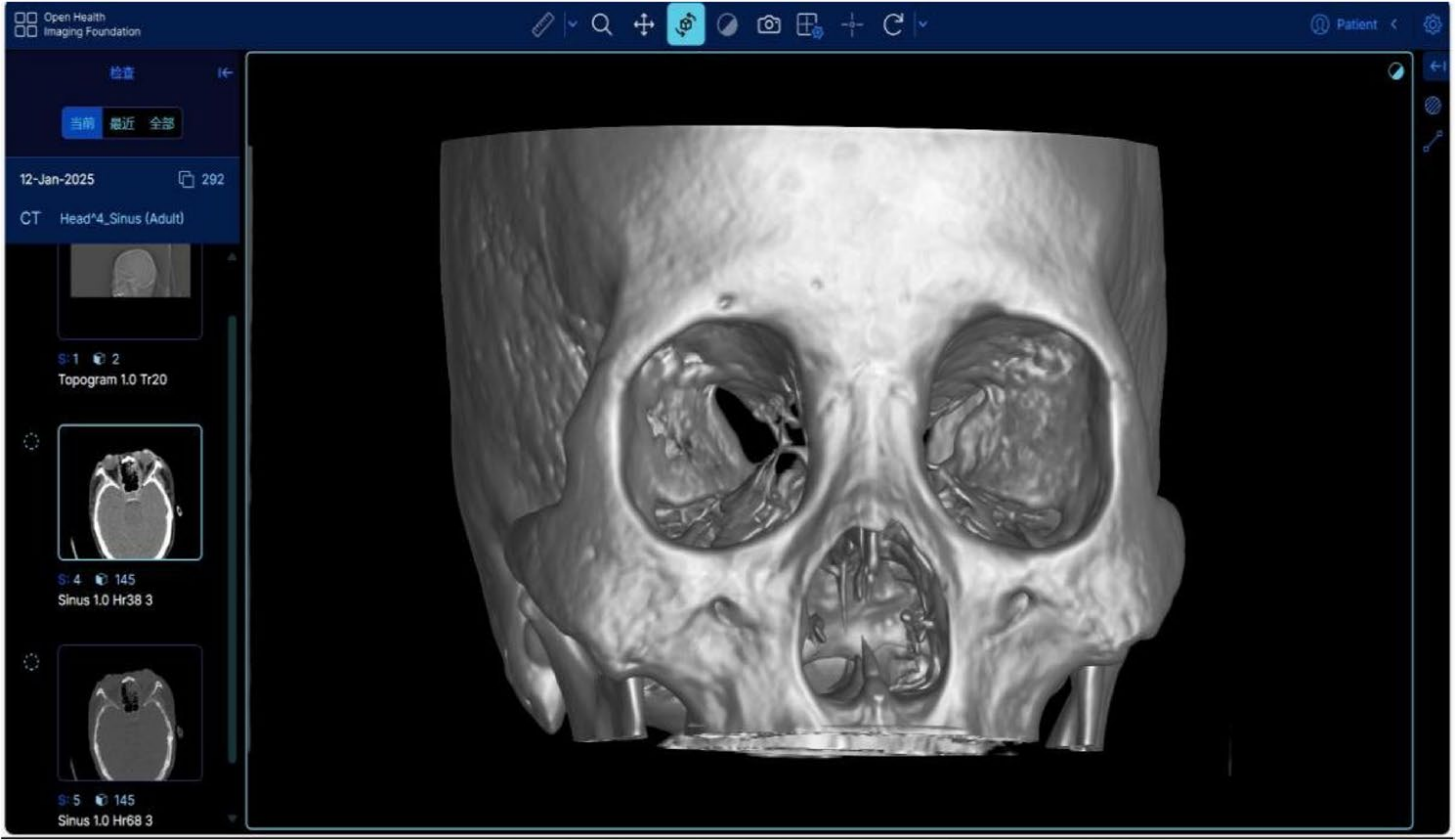
shows the DICOM data of case 1 obtained through the Orthanc connection to the PACS system. The interface is a four-window display, with scout images, bone window images, soft tissue window images from top to bottom on the left, and three-dimensional reconstructed frontal images on the right

CT data were acquired using a Siemens SOMATOM Drive dual-source CT scanner (120 kV tube voltage, 100 mA tube current) with patients in standard ocular scanning position. The data is transmitted to Orthanc through the hospital PACS system via the DICOM interface. The Orthanc interface receiving the data is shown in Fig. 2. Its image presentation mode is consistent with the terminal of the hospital PACS system, so it is saved as a DICOM file and imported into Mango for teaching.

### 3D model and simulation​

**Fig. 3:**
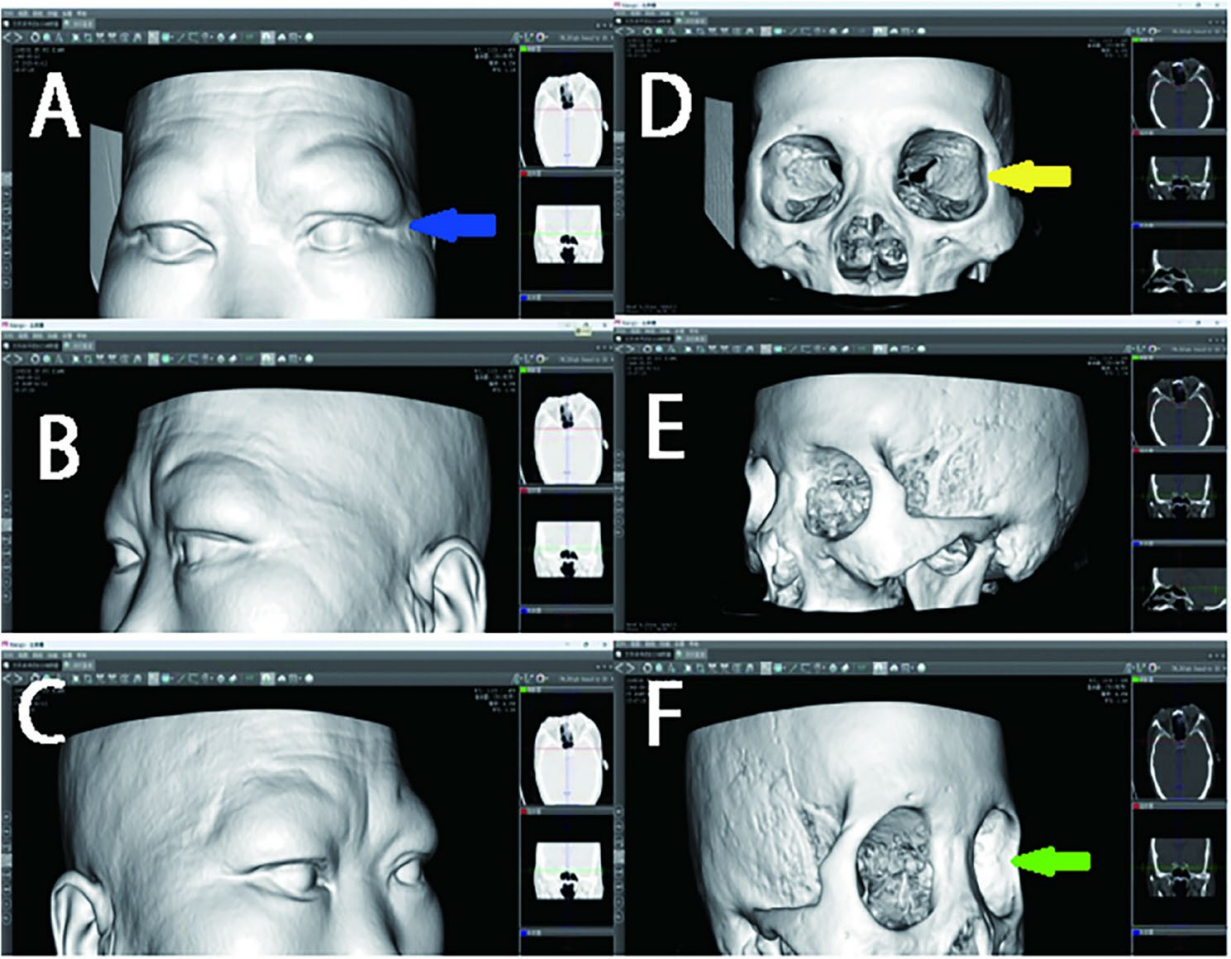
Three-dimensional reconstruction of Case 1 in Mango software. (**A**–**C**) Soft tissue reconstruction: (**A**) frontal, (**B**) left lateral, (**C**) right lateral views. (**D**–**F**) Bone tissue reconstruction: (**D**) frontal, (**E**) left lateral, (**F**) right lateral views. Key anatomical structures are indicated: orbital rim (yellow arrow), globe (blue arrow), and Orbital lateral wall (green arrow)

**Fig. 4:**
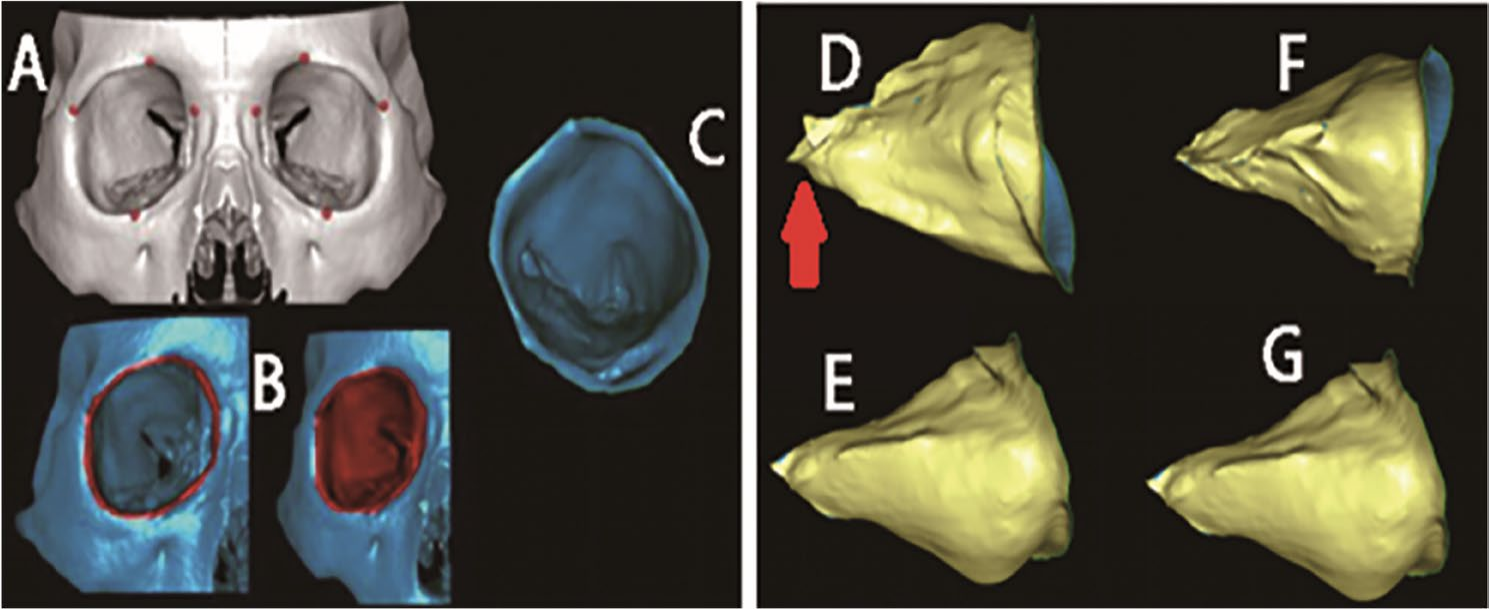
Process of orbital volume measurement for Case 1 using Mango software. (**A**) Initial placement of annotation points (red crosses) to define the orbital boundaries on an axial CT slice. (**B**) Results of the semi-automated segmentation: the right panel shows the defined orbital boundary (red outline), and the left panel displays the fully segmented orbital volume (blue overlay). (**C**) Three-dimensional reconstruction of the segmented right orbit, demonstrating the volumetric ROI(region of interest) used for calculation. (**D**-**G**) Multi-angle visualization of the 3D orbital structure: (**D**) right lateral view, (**E**) left lateral view, (**F**) view rotated 90 degrees horizontally from D, and (**G**) view rotated 30 degrees vertically from E. The orbital cavity is marked in yellow on the outer side, and the optic canal is labeled with a red arrow

**Fig. 5:**
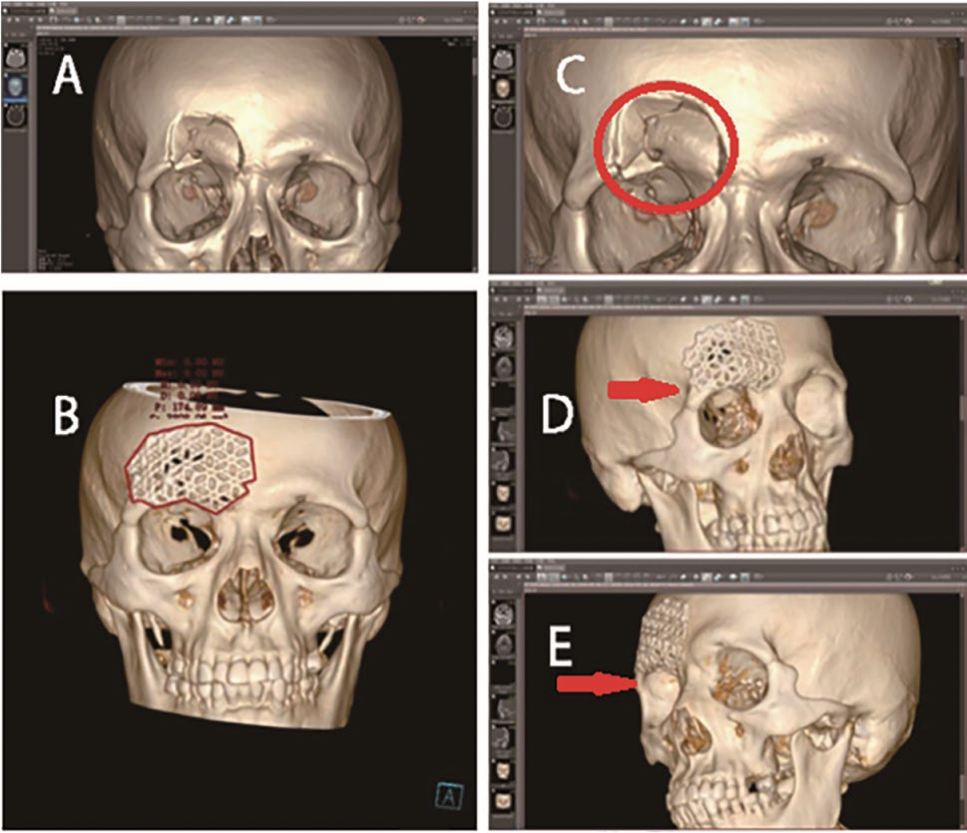
Preoperative views: (**A**) Frontal view of the 3D reconstruction showing the left orbital floor fracture. (**B**) Frontal view after reconstruction of the bone defect. The reconstructed area is outlined by a red line. (**C**) Enlarged frontal view of the fracture site, clearly displaying the bone defect (defect area indicated by red circle). Postoperative views: (**D**) Right lateral view of the postoperative model. (**E**) Left lateral view of the postoperative model, showing the restored contour of the orbital wall (red arrow)

On the same computer, the data obtained by Orthanc can be loaded into Mango software. By adjusting the window width and level settings, a three-dimensional reconstruction image of the patient’s soft tissue and bone tissue can be generated (see Fig. 3). This three-dimensional imaging data serves as a 3D model for surgical simulation, enabling multi-plane visualization of the orbital structure. During the surgical simulation, tools for segmentation, rotation, and tissue movement are used to simulate osteotomy, repositioning, and repair processes. The volume measurement of the orbit is achieved through anatomical landmark positioning, region extraction, and volume calculation [26]. To create a physical model, the segmented data is exported as an STL file by Orthanc, processed in Geomagic Studio 12.0, and then input into a CNC (Computer Numerical Control) machine for 3D printing to create a haptic model for practical training. Figure 4 shows the STL file of the orbit segmented using 3D image processing technology, which can be directly used for 3D printing of the orbital structure. Figure 5 displays preoperative planning (including defect quantification and implant design) and postoperative effect evaluation in digital form. Preoperatively, the 3D model can be used for surgical planning and preoperative design, including the position and size of implants. Postoperatively, the repair effect of the bone surgery can be evaluated from various angles, allowing for a comprehensive assessment of the surgery [27].

### Data analysis

After obtaining informed consent, fifty-eight clinical medicine students from the 2022 cohort at Haiyuan College, Kunming Medical University, were recruited as subjects and randomly allocated to an experimental group and a control group (29 students per group). The teaching period spanned one semester. Academic performance was evaluated through daily, mid-term, and final exam scores, with the control group receiving conventional teaching and the experimental group undergoing computer-aided surgical training. Daily scores were calculated using metrics from Rain Classroom (The online teaching platform of Haiyuan College) including pre-class preparation, post-class study duration, homework completion, attendance, and in-class participation. Mid-term and final exams were closed-book assessments using randomly selected questions from the ophthalmology question bank published by People’s Medical Publishing House, administered synchronously to both groups. The Surgical Training covered four modules: orbital fracture repair, eye socket reconstruction, diagnosis of ophthalmic plastic disorders, and teaching satisfaction. The first three modules were assessed via teacher-led practical training evaluations, while satisfaction was measured through a satisfaction questionnaire. Teaching satisfaction was evaluated using a validated weighted questionnaire (Supplementary File 1), administered post-intervention. The teaching satisfaction questionnaire was pilot-tested with a group of 10 students not involved in the study and reviewed by three expert ophthalmology educators to ensure content validity and clarity. Group-specific instruments captured technology experience, learning outcomes, and educational value for the CAST cohort, while the control group assessed traditional methods’ efficacy and limitations. Total scores (100-point scale) were derived from dimension-weighted formulas. All scores were recorded on a 100-point scale. Data were analyzed using SPSS 29.0. Independent sample t-tests were performed to compare group means, with t-values and P-values reported to determine statistical significance (two-tailed test). A post-hoc power analysis was conducted using GPower 3.1, which indicated that the sample size (*n* = 58) provided over 95% power to detect large effect sizes (Cohen’s d > 0.8) at α = 0.05, confirming the trial’s adequacy to identify the observed differences.

## Results

**Fig. 6:**
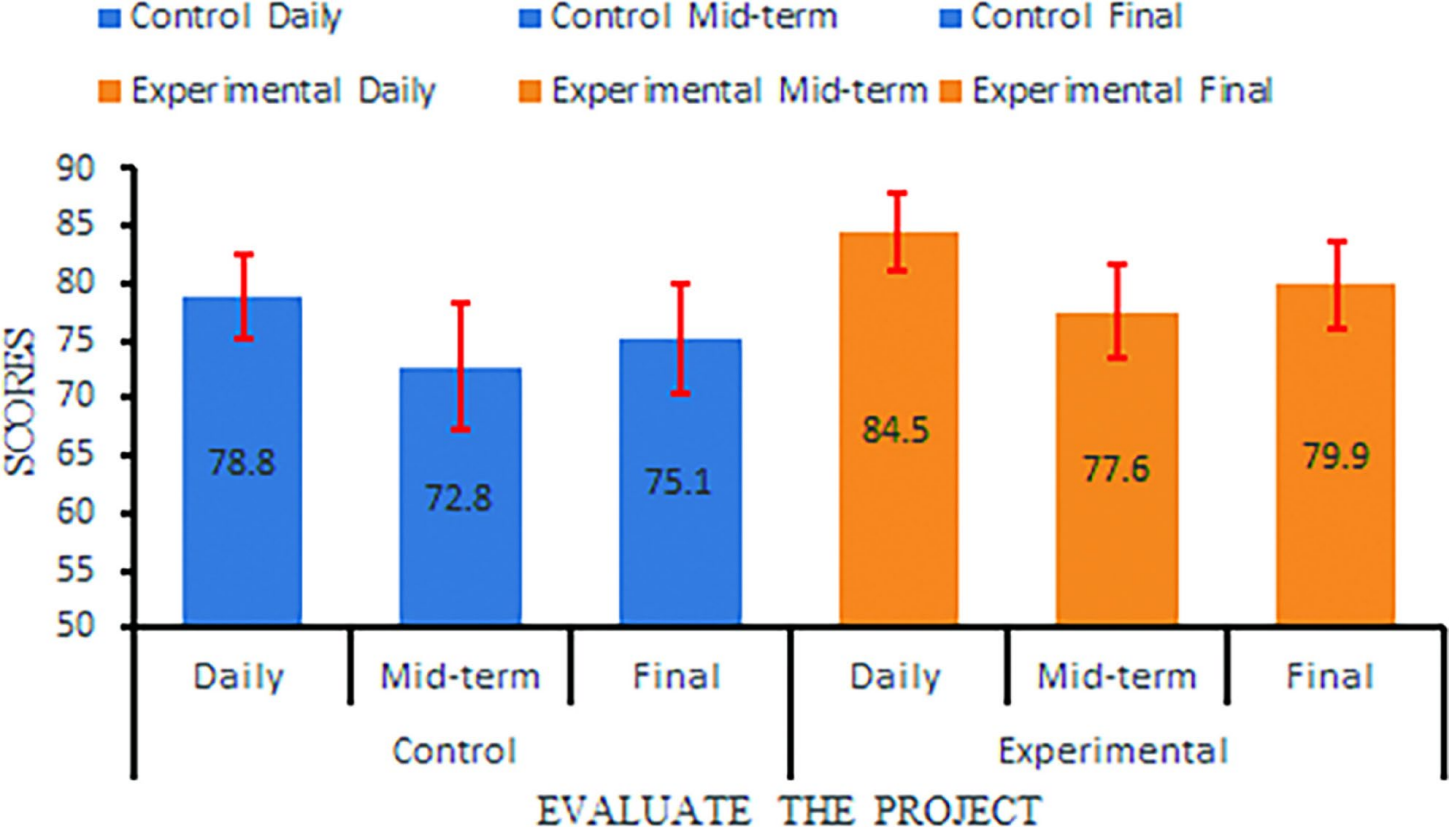
Comparison of theoretical test scores between experimental group and control group: it is divided into three items: daily score, midterm score and final score. The horizontal coordinate represents different items, and the vertical coordinate is the score (100-point system). Blue corresponds to the control group, and orange corresponds to the experimental group

The experimental group outperformed the control group in all theoretical and surgical training assessment metrics. Independent sample t-tests (two-tailed) revealed statistically significant differences (all *P* < 0.001), with large effect sizes as measured by Cohen’s d, as summarized in Table 1; Fig. 6. Specifically, the experimental group demonstrated superior performance in:

Theoretical Knowledge: Daily, mid-term, and final exam scores were significantly higher (*P* < 0.001 for all comparisons), with large effect sizes (Cohen’s d = 1.604, 0.981, and 1.097, respectively).

**Table 1:** Comparison of theoretical assessment scores between the control and experimental groups

Group (*n* = 29 per group)	Daily Performance (Mean ± SD)	Mid-term Exam (Mean ± SD)	Final Exam (Mean ± SD)
Control	78.8 ± 3.7	72.8 ± 5.5	75.1 ± 4.8
Experimental	84.5 ± 3.4	77.6 ± 4.2	79.9 ± 3.9
t-value	−5.94	−3.84	−3.72
*P*-value	< 0.001	< 0.001	< 0.001
Cohen’s d (95% CI)	1.604 (1.012, 2.196)	0.981 (0.436, 1.526)	1.097 (0.545, 1.649)

Effect sizes are reported as Cohen’s d with 95% CI

IntervalIndependent samples t-test (two-tailed) was used for comparisons

The negative t-value indicates that the experimental group scored higher than the control group

*SD* Standard Deviation, *CI* Confidence

### Surgical training

Assessment scores for orbital fracture repair, eye socket reconstruction, and diagnostic accuracy of ophthalmic plastic disorders were all statistically higher (*P* < 0.001 for all).

### Teaching satisfaction

**Fig. 7:**
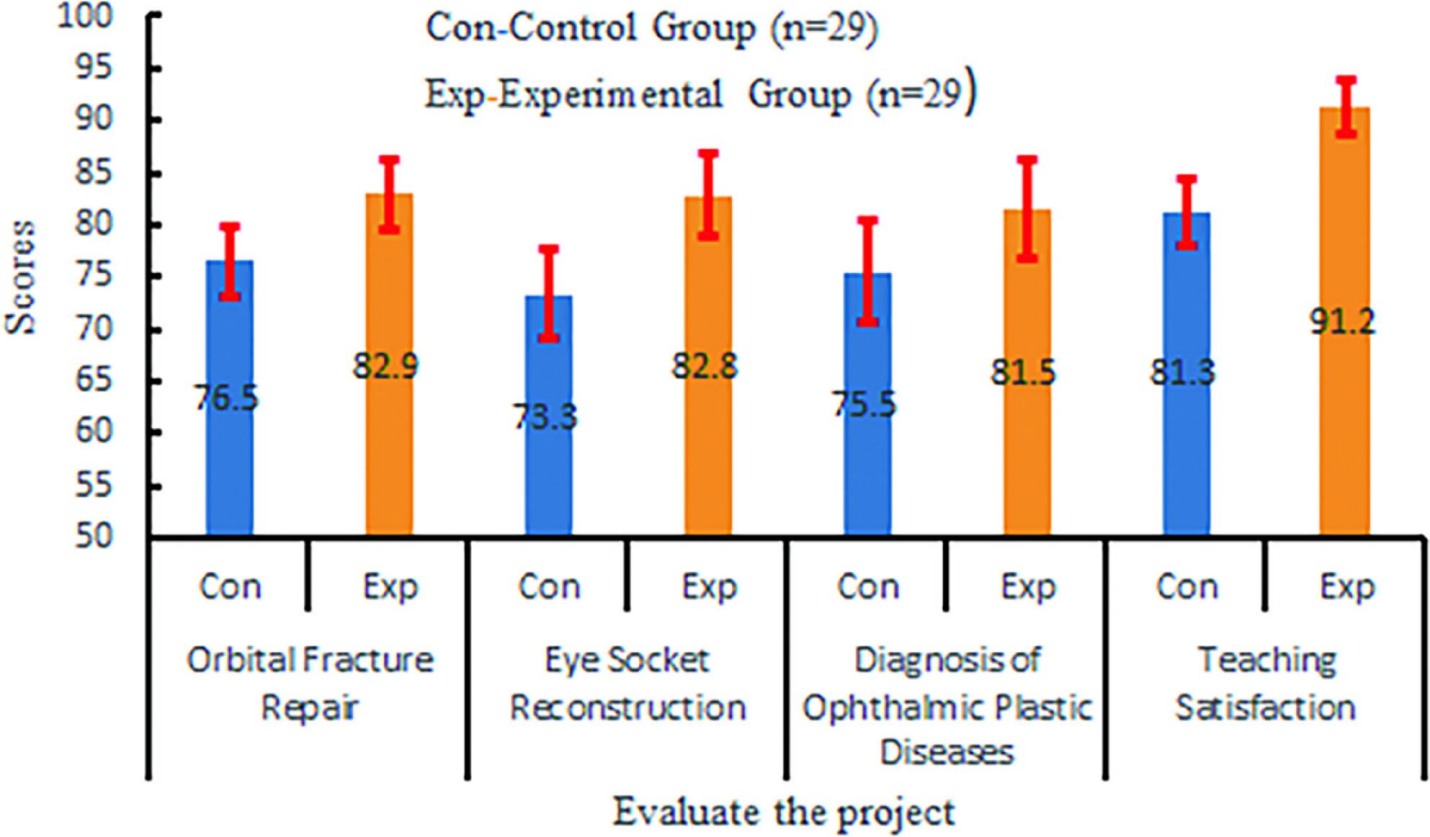
Compares the results of three practical training and one satisfaction survey between the experimental group and the control group. The horizontal axis represents different items, and the vertical axis is the score (100 points system). Blue corresponds to the control group, and orange corresponds to the experimental group

The experimental group reported a satisfaction rate of 91.24, nearly 10% points higher than the control group’s 81.31(*P* < 0.001), as shown in Table 2; Fig. 7.

**Table 2:** Comparison of practical skill assessment scores and teaching satisfaction between the control and experimental groups

Skill Domain	Control Group (Mean ± SD)	Experimental Group (Mean ± SD)	t-value	*P*-value	Cohen’s d (95% CI)
Orbital fracture repair	76.5 ± 3.3	82.9 ± 3.3	−7.35	< 0.001	1.939 (1.315, 2.563)
Eye socket reconstruction	73.3 ± 4.3	82.8 ± 4.0	−8.75	< 0.001	2.287 (1.625, 2.949)
Diagnostic accuracy	75.5 ± 4.9	81.5 ± 4.8	−4.74	< 0.001	1.237 (0.675, 1.799)
Teaching satisfaction	81.3 ± 3.2	91.2 ± 2.5	−13.17	< 0.001	3.448 (2.636, 4.260)

Independent samples t-test (two-tailed) was used for all comparisons

Effect sizes are reported as Cohen’s d with 95% CI

*SD* Standard Deviation, *CI* Confidence Interval

## Discussion

Traditional ophthalmic surgery education relies heavily on didactic teaching, with few opportunities for hands-on experience—particularly for large classes due to ethical and resource constraints. CAST addresses this gap by enabling preoperative planning, procedural rehearsal, and outcome evaluation in risk-free digital environments. Our study pioneers an accessible CAST workflow leveraging Orthanc (DICOM server) and Mango (3D segmentation) to replicate hospital PACS-to-simulation pathway on standard PCs. The key innovations and findings of our study are as follows. First, in terms of educational efficacy, the experimental group demonstrated statistically superior performance in theoretical knowledge (P<0.001), practical surgical skills (e.g.+9.49 points in orbital reconstruction; P<0.001), and satisfaction (91.2 vs. 81.3; P<0.001). This aligns with evidence that 3D visualization improves spatial understanding of complex anatomy [28–30].

When contextualized within the landscape of existing simulation modalities, the strengths of our low-cost, open-source workflow become apparent. Compared to VR systems, which offer high immersion but incur substantial costs (often exceeding US$50,000) and require specialized hardware, our solution provides accessible 3D visualization and planning on standard computers at a fraction of the cost. While cadavers remain the gold standard for tactile fidelity and true tissue handling, they are associated with ethical considerations, high expenses, limited availability, and inability to rehearse patient-specific pathology. Commercial surgical planning software (e.g., Materialise Mimics) offers robust functionality but involves significant licensing fees, creating barriers for widespread educational adoption. The workflow presented here, leveraging Orthanc and Mango, strikes a critical balance: it offers superior spatial understanding compared to 2D methods and traditional lectures, introduces patient-specific planning unlike static models, and maintains high accessibility and scalability unattainable by VR, cadaveric, or commercial alternatives. This makes it particularly suitable for foundational surgical education in resource-conscious environments. The cost-effectiveness and accessibility of this approach directly address some of the key barriers, such as high costs and specialized equipment requirements, that have been identified as major obstacles to the adoption of 3D printing and advanced simulation technologies in medical education [31].

The early exposure to structured surgical planning and rehearsal provided by our CAST framework is hypothesized to foster better habits of preoperative preparation and spatial reasoning. If sustained, these foundational competencies could subsequently translate into improved clinical performance during residency, potentially leading to enhanced patient safety through reduced procedural errors and improved surgical quality. Future tracking of participants into their postgraduate training would be invaluable to substantiate this potential cascade from educational intervention to ultimate clinical impact.

The significant improvement in theoretical scores (6.7–8.2 points) aligns with cognitive principles such as dual coding theory, where 3D visualization reinforces learning through visual and motor channels [32–34]. Similarly, tactile feedback from 3D-printed models supports embodied cognition, enhancing practical skill acquisition, as reflected in the 9.5-point increase in eye socket reconstruction scores in Table 2.

The open-source tool-chain established in this study demonstrates significant clinical educational adaptability: Orthanc server’s simulation of hospital PACS systems aligns the teaching scenario with clinical workflows, leading to an 89.7% diagnostic coincidence rate in postoperative CT assessment tasks for the experimental group, a 14.2% improvement over the control group [35]. Notably, surface treatment techniques for 3D-printed models (e.g., smoothing and landmark generalization) ensure anatomical accuracy while controlling patient identification risk [33] below 0.1% (ISO/TS 25237 standard), providing technical guarantees for ethically compliant teaching practices. This scheme has been promoted in The First People’s Hospital of Yunnan Province, reducing preoperative planning time for ophthalmic residents by 40% (from 2.5 to 1.5 h on average).

Workflow portability: By transitioning surgical simulation from operating rooms to classrooms, our solution resolves the conflict between clinical service demands and educational needs—a critical advantage for resource-constrained institutions. Cognitive benefits: Interactive case-based simulation promotes deeper learning through deliberate practice, correlating anatomical variants with surgical decision-making [36].

Furthermore, the workflow can be extended to directly utilize 3D printing technology to produce physical teaching models, transforming the virtual teaching process into physical objects. The 9.9 satisfaction gap reflects CAST’s ability to bridge the theory-practice chasm in surgical education. By converting abstract concepts into tactile 3D models, students achieve cognitive integration of spatial anatomy—a critical step in competency-based curricula [37]. This simulation approach aligns with Kolb’s experiential learning theory, as concrete experience through 3D modeling and active experimentation through surgical simulation accelerate skill internalization.

While the significant improvements in theoretical exam scores and structured practical assessments indicate a strong foundation of knowledge and procedural understanding, we acknowledge that these measures, combined with satisfaction surveys, are proximal indicators of learning. They primarily reflect skill acquisition under controlled conditions. True surgical competency encompasses integration of knowledge, technical skill, clinical reasoning, and non-technical skills, which often require longer-term evaluation in clinical settings. The practical assessments used in this study, particularly the simulated orbital fracture repair and socket reconstruction tasks, were designed to mirror key steps of actual procedures (e.g., anatomic recognition, planning, and bimanual manipulation), thereby providing a valid proxy for initial technical skill acquisition. However, to more comprehensively evaluate the translation of these skills into clinical practice and their long-term retention, future studies should incorporate longitudinal follow-up assessments during students’ clinical rotations or residency training.

### Limitations and future directions

This study has several limitations that should be considered. First, the assessment of skill acquisition in our methodology relied primarily on immediate post-intervention evaluations, which may not fully reflect long-term knowledge retention or clinical skill transfer. Second, our sample was drawn from a single institution, limiting the generalizability of our findings [34]. Third, the current simulation models lack dynamic physiological parameters, such as extraocular muscle tension, which could be addressed through finite element analysis for enhanced biomechanical modeling [38]. Fourth, the workflow has not yet incorporated AI-assisted surgical decision support, whereas recent evidence shows CNN-based systems can improve surgical plan rationality [39].

To address these limitations, our future research will focus on three key directions: (1) conducting longitudinal follow-up studies to assess skill retention and clinical application during residency training; (2) enhancing model realism through the integration of finite element analysis to simulate biomechanical properties [38]; and (3) developing AI-powered surgical planning modules, including CNN-based recommendation systems for implant sizing and osteotomy planning [39]. Additionally, we plan to establish a multicenter evaluation network to validate the educational efficacy of this approach across diverse institutional settings [34].

## Conclusion

This study establishes a feasible, low-cost CAST framework using open-source tools (Orthanc/Mango) and 3D printing to enhance ophthalmic plastic surgery education. Key contributions include: Pedagogical innovation: Bridging theory-practice gaps through immersive simulation, significantly improving knowledge retention (*P* < 0.001) and Surgical Training competency. Operational scalability: The standalone workflow enables institution-wide deployment without disrupting clinical operations. Evidence-based prompt: Quantifiable gains in diagnostic accuracy (+ 6.0 points) and procedural skills indicate CAST as a critical adjunct to traditional teaching. Future work should focus on AI-enhanced simulation personalization and competency-based progression metrics.

## Supplementary Information

Supplementary Material 1.

Supplementary Material 2.

## Data Availability

The datasets used and analysed during the current study are availablefrom the corresponding author on reasonable request.

## Abbreviations

CAST: Computer-assisted surgery technology
DICOM: Digital imaging and communications in medicine
CT: Computed tomography
MRI: Magnetic resonance imaging
PACS: Picture archiving and communication systems
VR: Virtual reality
STL: Stereo lithography
CNC: Computer numerical control
ROI: Region of interest
