# Supplementary material for: Research on the application of computer-assisted surgical technology in ophthalmic plastic surgery education​
Source: BMC Med Educ. 2025 Dec 22;26:137. doi: 10.1186/s12909-025-08158-8 (PMC12837335; doi:10.1186/s12909-025-08158-8)
Supplement: Supplementary file 1 — Supplementary Material 1. [file 12909_2025_8158_MOESM1_ESM.pdf]

Satisfaction Questionnaire for Computer-Assisted Ophthalmic Plastic Surgery Education

(Dual-Group Version for Randomized Controlled Trial)

I. Experimental Group (CAST-Based Teaching)

Instructions: Rate your agreement with each statement using the 10-point scale, where:
1 = Strongly Disagree ——— 10 = Strongly Agree

Dimension & Weight	Item	Rating (1-10)
**I. Technology Experience (Weight: 30%)**	1. Orthanc DICOM retrieval realistically simulated clinical PACS workflows	□
	2. Mango's 3D segmentation interface was intuitive for orbital tissue isolation	□
	3. Virtual surgical manipulations (e.g., osteotomy) provided sufficient realism for skill training	□
	4. 3D-printed models offered valuable haptic feedback during rehearsal	□
**II. Learning Outcomes (Weight: 35%)**	5. Enhanced understanding of complex spatial anatomy (e.g., orbital volume calculation)	□
	6. Improved surgical planning competence (e.g., implant positioning)	□
	7. Virtual outcome evaluation improved insight into surgical success criteria	□
**III. Educational Value (Weight: 25%)**	8. This methodology is more effective than traditional teaching for skill acquisition	□
	9. I intend to apply CAST technology in my future clinical practice	□
**IV. Global**	10. Overall satisfaction (including hardware, software, and	□

Dimension & Weight	Item	Rating (1-10)
**Assessment (Weight: 10%)**	curriculum design)	

Scoring Formula:
Total Score = [(Mean of Items 1-4) × 0.30 + (Mean of Items 5-7) × 0.35 + (Mean of Items 8-9) × 0.25 + Item 10 × 0.10] × 10

II. Control Group (Traditional Teaching)

Instructions: Rate your agreement with each statement using the 10-point scale, where:
1 = Strongly Disagree ——— 10 = Strongly Agree

Dimension & Weight	Item	Rating (1-10)
**I. Teaching Tool Efficacy (Weight: 30%)**	1. 2D atlases/videos adequately demonstrated orbital anatomy	☐
	2. Instructor demonstrations effectively guided procedural training	☐
	3. Cadaveric specimens/static models facilitated spatial understanding	☐
**II. Instructional Limitations (Weight: 35%)**	4. Inability to analyze patient-specific DICOM data restricted learning depth	☐
	5. Absence of surgical planning simulation reduced decision-making practice	☐
	6. Difficulty visualizing postoperative outcomes (e.g., bone defect repair)	☐
**III. Learning Outcomes (Weight: 25%)**	7. Confidence in performing standardized surgical procedures	☐
	8. Understanding spatial relationships of anatomical variations	☐
**IV. Reform Needs (Weight: 10%)**	9. Traditional methods have significant limitations for complex surgery training	☐
	10. I support integrating CAST technology into the curriculum	☐

Scoring Formula:
Total Score = [(Mean of Items 1-3) × 0.30 + (Mean of Items 4-6) × 0.35 + (Mean of Items 7-8) × 0.25 + (Mean of Items 9-10) × 0.10] × 10

Administration Protocol

Timing: Administered immediately post-curriculum (≤5 minutes completion time)

Anonymity: No personal identifiers collected

Raw scores converted to weighted 100-point totals per formula
