# Supplementary figures and images for: Research on the application of computer-assisted surgical technology in ophthalmic plastic surgery education​
Source: BMC Med Educ. 2025 Dec 22;26:137. doi: 10.1186/s12909-025-08158-8 (PMC12837335; doi:10.1186/s12909-025-08158-8)

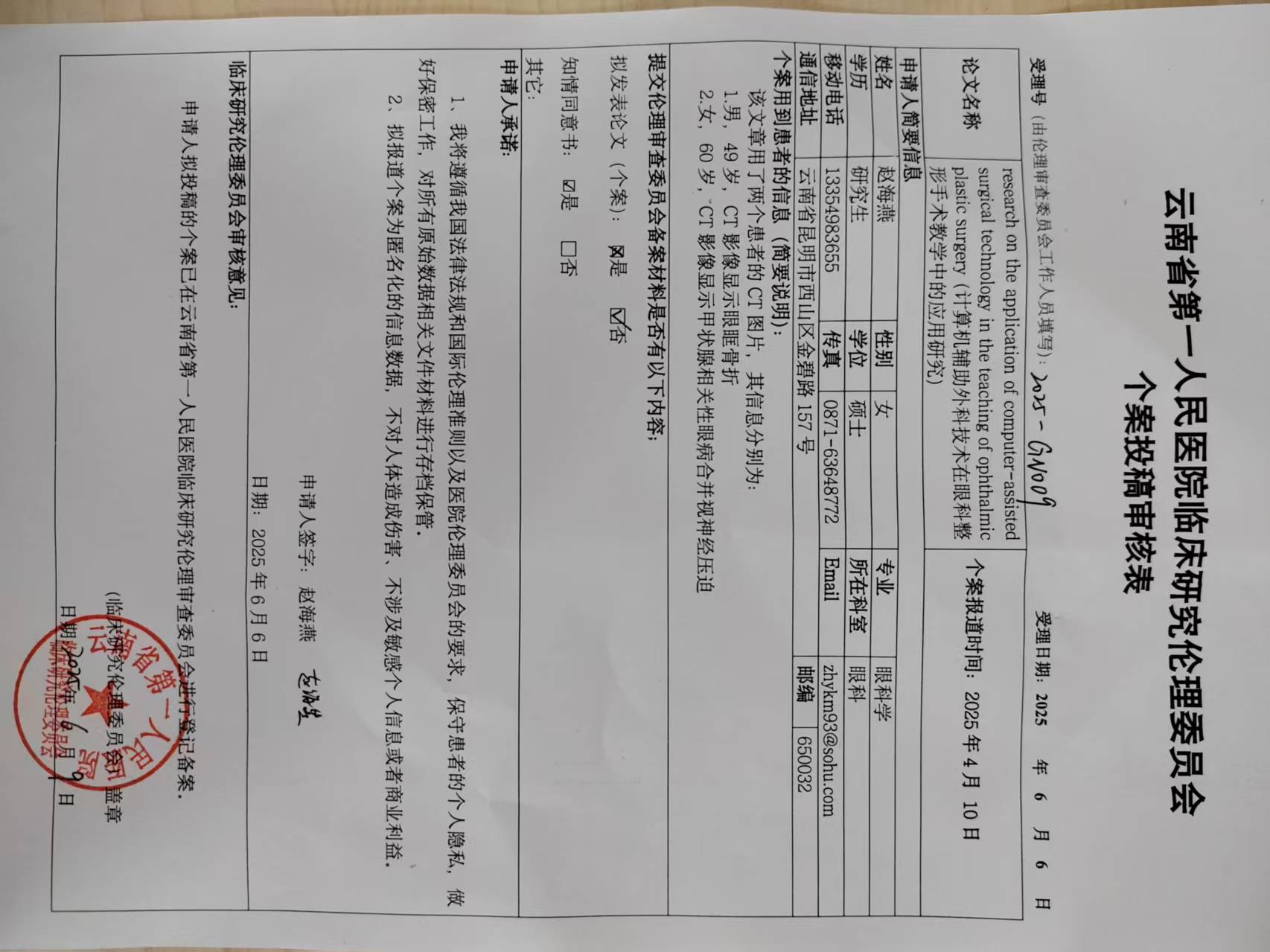

Supplement: Supplementary file 2 — Supplementary Material 2. [file 12909_2025_8158_MOESM2_ESM.jpg]
